# Women’s Experiences with Exercise-Induced Orgasm: Findings from Qualitative Interviews

**DOI:** 10.1007/s10508-025-03237-9

**Published:** 2025-10-02

**Authors:** Debby Herbenick, Callie Patterson Perry, J. Dennis Fortenberry, Ruhun Wasata, Jodi Wilson, Owen Miller, Kayla Willens, Alyssa Williams, Georgia Frey

**Affiliations:** 1 The Center for Sexual Health Promotion, Indiana University School of Public Health, Indiana University, Bloomington, IN 47401 USA; 2 Department of Applied Health Science, Indiana University School of Public Health, Indiana University, Bloomington, IN USA; 3 Department of Public Health, College of Health Sciences, Des Moines University, West Des Moines, IA USA; 4 Department of Pediatrics, Indiana University School of Medicine, Bloomington, IN USA; 5 Department of Kinesiology, Indiana University School of Public Health, Indiana University, Bloomington, IN USA

**Keywords:** Orgasm, Exercise-induced orgasm, Sexual pleasure, Sexual development, Arousal

## Abstract

Exercise-induced arousal (EIA) and exercise-induced orgasm (EIO) have been described as occurring from engaging in exercise or physical activities that are vigorous, repetitive, or demanding of core abdominal muscles, usually apart from sexual situations and without direct genital stimulation. Few existing studies have examined EIA/EIO, and no prior studies have qualitatively explored these experiences. Thus, the purpose of this research was to understand women’s lived experiences of EIO. We used a qualitative semi-structured interview format to ask women to describe when and under what circumstances they first experienced EIO, their understanding of how EIO feels to them and occurs in their body, the exercises that lead to their EIO experiences, as well as whether they feel EIO may be similar to or different from other orgasms they have experienced. A total of 21 adult women ages 19–68 years participated in in-person (*n* = 18) or virtual (*n* = 3) qualitative interviews. Data were coded into the following six categories: Discovering and Making Sense of EIO; How EIO is Experienced; Factors Associated with EIO; Communication about EIO; Feelings About EIO; and Intersection Between EIO, Sex, and Masturbation. Many participants described first experiencing EIA/EIO during childhood or adolescence. They reported EIA/EIO from diverse exercises (core exercises, swimming, strength training, yoga, among others) and varied in the extent to which they had discussed their EIA/EIOs with friends, partners, or healthcare providers. Although some felt embarrassed or ashamed about EIA/EIO, others felt positively about their experiences and, in some cases, even incorporated their experiences into partnered sexual experiences.

## Introduction

Orgasm is typically described as arising from genital and/or sexual stimulation (Meston et al., 2004) and as occurring throughout the life course (Bancroft et al., 2003; Fugl-Meyer et al., 2006; Robbins et al., 2011). Orgasms from solo masturbation are often situated within people’s sexual and genital exploration as they learn how they respond to sexual stimuli and map the kinds of emotional states, thoughts, touches (genital as well as other body areas), and actions that they come to associate with their arousal, pleasure, and orgasm (Barbach & Ayres, 1976; Rowland et al., 2020). For some, part of this sexual learning may involve noticing that not all orgasms occur due to sexual activity or genital contact, inviting consideration of orgasm in more expansive ways (Herbenick et al., 2018). Indeed, orgasm is known to occur in response to non-sexual activities, both with and without genital stimulation; these include sleep, exercise, drug use, meditation, nursing, birthing, listening to music, and eating certain foods (Crossing, 2021; Herbenick et al., 2011, 2018; Kenny, 1973; Komisaruk & Whipple, 2011).

Exercise-induced orgasm (EIO) tends to occur while engaging in exercise or physical activities that are vigorous, repetitive, and/or demanding of core abdominal muscles; EIO usually occurs apart from sexual situations and often without direct genital touch or external stimulation (Herbenick & Fortenberry, 2011; Herbenick et al., 2021). Although briefly noted by Kinsey et al., (1948, 1953) and, later, in case reports and media articles (the latter often using the term “coregasm”; Barnes, 2016), the first systematic study of EIO did not occur until Herbenick and Fortenberry (2011) surveyed a convenience sample of 530 women about their experiences with exercise-induced arousal (EIA) and EIO. In this study, more than half of women described feeling generally happy with their EIO experiences but most reported feeling, at least some of the time, embarrassment related to EIO (Herbenick & Fortenberry, 2011). About 80% of women reported having worried that others might notice their EIA/EIO experience (such as from their facial expressions or vocalizations), highlighting the potentially negative assessment of EIO occurring in group exercise spaces such as gyms or physical education classes. Self-consciousness or embarrassment may be heightened by the fact that less than one-third of women in this survey felt that they could control their EIO experiences, reflecting that EIOs tended to occur spontaneously and were difficult to suppress. It is not clear to what extent EIA and EIO are part of a spectrum of experience or whether they may be distinct phenomenon.

In a US nationally representative survey of adolescents and adults, lifetime EIO was reported by about 10% of women and 8% of men (Herbenick et al., 2021). Although neither the convenience survey nor the US nationally representative survey was designed to examine potential mechanisms for EIO, each study found that EIO occurs most frequently in connection with repetitive exercises that engage the core abdominal muscles, such as abdominal crunches, sit-ups, and Roman chair leg raises. Herbenick et al., (2021) also examined EIO in relation to orgasms occurring during sleep—another type of orgasm that challenges ideas of orgasm being an innately sexual experience. In that study, women who had ever experienced EIO were significantly more likely to report having experienced sleep orgasm. However, there was no relationship between a prior history of EIO and the likelihood of experiencing orgasm at one’s most recent partnered sexual event. Aside from this one study, no existing research has assessed how EIO experiences may be situated in the context of women’s experience of orgasms during solo or partnered sexual experiences.

In summary, there have only been two published studies that focus on EIA and EIO and both have been quantitative in nature. Little is known about how people experience EIA/EIO, especially given that these experiences may occur in public spaces. We don’t know, for example, how people feel about having EIO in public or how it feels for their body and orgasmic response to feel beyond their control. Further, given that the research on EIA/EIO is only beginning to emerge, there is currently no known mechanism for understanding how EIA/EIO occur. Toward this goal, it will be important to gather people’s detailed descriptions of the exercises that lead to their EIA/EIO as well as where in their body they begin to feel arousing and/or orgasmic sensations, as these might provide clues toward an eventual understanding of the processes that underlie EIA and/or EIO. Finally, although prior research has noted that some people recall having first experienced EIA/EIO during childhood or adolescence (Herbenick et al., 2021), it is not yet known what these experiences are like, how people make sense of these bodily responses during childhood, or how EIA/EIO may fit into normative sexual development. The present study addresses these gaps.

### Study Aims

We aimed to understand women’s lived experiences of EIO. Accordingly, we used a qualitative semi-structured interview format to ask women to describe when and under what circumstances they first experienced EIO, how EIO feels to them, their understanding of how it occurs in their body, exercises that lead to their EIO experiences, and how they consider EIO to be similar to or different from other orgasms they may have experienced.

## Method

### Participants

Recruitment messages were posted on a university website and shared through personal networks. Women were eligible to participate if they were at least 18 years old and had experienced orgasm from physical exercise. Participants received twenty-dollar gift cards at the completion of the interview.

### Measures and Procedure

A total of 21 women participated in the study (mean age = 26.9, SD = 10.9, range = 19–68); thirteen were between ages 19 and 25 (Table 1). Most were white and from the USA (Illinois, Indiana, Louisiana, Massachusetts, Missouri, New York, Wisconsin). Three were born outside of the USA (China, India, and Iran) and lived in the USA at the time of the interview. Most were currently physically active with a regular exercise routine. Also, most had been sexually active with a partner in the prior year.

**Table 1:** Participant characteristics

Participant ID	Age	Age at First EIO	Exercises That Cause EIO
1	25	5/6	Climbing vertical poles during childhood; within 5 min of continuous leg raises using chair
2	20	8/9	40–60 crunches; Certain yoga stretches and Pilates moves
3	19	15/16	Hanging leg raises (50–75 reps), knee ups (3 sets of 30), hanging abs (20–30 reps), and biking
4	19	15/16	Within 20 min of outdoor cycling
5	21	7	Candlestick pose (1 min), captains chair (1–2 sets of 10), climbing rope, after 3 consecutive minute-long planks
6	25	23	Abdominal exercises after running on treadmill/elliptical (1 h; 30 min incline); after 16 sit-ups
7	26	19/20	Spinning, 30–50 leg lifts, yoga (high or low boat pose)
8	31	20	Yoga (leg bicycles while holding V posture, holding warrior pose, triangle), sit-ups and other abdominal exercises (after 5 sets, or roughly 150–200 reps)
9	20	15/16	Biking and yoga
10	21	20	Indoor cycling (after around 50 min)
11	39	35	Captain’s chair, leg raises (after 2–3 reps of 10)
12	20	15/16	Sprinting, cycling, leg exercises (2 sets of 30–40 walking lunges, third set of 50 squats) and fire hydrants (after 2–3 sets of 15)
13	22	14	Swimming (10–15 min after warm-up)
14	21	18	Elliptical (after at least 30 min)
15	29	9	Climbing poles/ropes (after 30 s), parallel bars
16	34	14	After up to 100 captain’s chair leg raises, or leg raises in accessible bathroom stall, crunching abdominal exercises
17	23	12/13	Bar hang (1 min) or after 3–9 pull-ups
18	27	4/5	Monkey bars, roman chair (20 reps of knee raises, another 10 for subsequent orgasms), pull-up bar
19	68	8/9	Initially, carnival rides or swings. Later, horseback riding, motorcycle riding, gymnastics, sit-ups, yoga, and meditation
20	32	11	Cycling, elliptical (after 30 min)
21	22	16/17	Indoor and outdoor cycling (after around 2 h), abdominal exercises (flutter kicks, bicycles)

Prior to each interview, the researchers reviewed the IRB-approved Study Information Sheet with individuals. Verbal consent was obtained for study participation. Interviews were audio-taped, professionally transcribed, and checked for accuracy. Interviews lasted about one hour. Most were conducted in a private on-campus office by two women interviewers (a sexuality researcher and a graduate student who was a certified personal trainer). Three were conducted virtually for out-of-state participants. The semi-structured interview guide was developed by the first author and included open-ended questions about participants’ EIO experiences (exercises that led to EIO, how reliable their exercise orgasms were), their first EIO experiences, how their EIO experiences compared with other orgasms, disclosure of EIO to others as well as interpretations and feelings about EIO experiences. Interviews continued until the interviewers agreed that data saturation had occurred, when meaningfully new information no longer emerged from interviews, and information became redundant (Saunders et al., 2018). Interviews were audio-recorded and professionally transcribed.

The interview room was equipped with exercise equipment such as exercise mats, exercise balls, dumbbells, and a Roman chair. The equipment was available so that participants whose EIO/EIA had been produced by the types of materials available (e.g., who could conduct their exercise on a floor mat or with hand weights) could demonstrate, if they felt comfortable, how they engaged in the exercises that tended to produce EIO for them. The researchers explained that they were not asking for participants to exercise to the point of orgasm, but just to briefly demonstrate how they performed the exercise or moved their body. Indeed, orgasm would have been unlikely to occur, as EIO tends to occur after substantial repetitions of an exercise (e.g., 50–300 crunches) and the researchers asked participants to demonstrate, if comfortable, just a few repetitions in order to gain insights about body position and movement. Participants were also shown an anatomical poster that illustrated muscles throughout the body and were asked whether they could identify where they felt sensations of muscular tension or activation. Finally, participants were shown a rate of perceived exertion chart to help contextualize the intensity of the exercise that produced EIO/EIA. Information about exercise mechanics, muscle activation, and exertion was transcribed as part of the recorded interviews but is beyond the scope of this paper, which is focused on women’s experiences with and feelings about EIA/EIO.

### Analysis

The analytic team was led by a senior level doctoral student with experience conducting sexuality research and qualitative research, with support from a faculty researcher with two decades of sexuality research experience. The coding was conducted by three doctoral students and two undergraduate research assistants, each of whom was experienced with sexuality research. The analytic process was guided by the Braun and Clarke’s (2006) iterative six-step process for thematic analysis: (1) familiarization, (2) code generation, (3) searching for themes, and (4) reviewing, (5) defining, and (6) reporting themes.

The research team engaged in a process of inductive codebook generation which involved creating a codebook based on the themes and patterns observed from the data. To accomplish this, each coder individually read the first five interviews to familiarize themselves with the data. The two lead authors then met with the full group to train research assistants in coding. Next, coders were paired up. The first pair was responsible for coding the first three odd-numbered interviews and the second pair was asked to code the first three even-numbered interviews. Each member coded interviews independently, then compared and coordinated codes with their coding partner. The full group then met to discuss and coordinate codes, areas of disagreement in codes, and reach a consensus on codes. A full codebook was created to help guide the analysis of the remaining interviews. Previously coded data were checked to ensure that all codes had been properly applied.

The pairs then coded the remaining interviews using the completed codebook. Emergent codes were added when identified, and consensus coding meetings occurred within pairs to integrate emergent codes into the codebook and discuss any problems with applying the codebook to the data. Throughout the analytic process, there were two additional full-team coding coordination and consensus meetings, led by the doctoral student research coordinator. One pair coded 11 interviews; the second pair coded 10 interviews. Each interview was coded by at least two research assistants. This iterative process created an opportunity for peer debriefing, thorough discussion, understanding, and team agreement on the coded data, increasing trustworthiness of the study (Macdonald et al., 2019; Nowell et al., 2017).

## Results

Interview data were coded into the following categories: Discovering and Making Sense of EIO (first experiences, understanding EIO, intentional engagement with EIO); How EIO is Experienced (how it feels, location of sensations, comparison with other sexual sensations; EIO frequency); Factors Associated with EIO (exercises, clothing/accessories, menstruation/menopause); Communication about EIO; Feelings About EIO (embarrassment, shame, distraction, enjoyment/acceptance); and Intersection Between EIO, Sex, and Masturbation.

### Discovering and Making Sense of Exercise-Induced Arousal/Exercise-Induced Orgasm

Women described memories of their first EIA/EIO experience(s), including the exercises that first produced EIA or EIO, how their body felt, and how they understood their bodily sensations. Some described repeatedly returning to the exercise to reproduce the feelings.

#### First Experiences with Exercise-Induced Orgasm

Most women described having their first EIA/EIO experience during childhood (as young as age four or five) or early adolescence (see Table 1). Four participants indicated that their first EIO experience occurred during adulthood. Those whose first EIA/EIO occurred at younger ages often described curiosity, bewilderment, or confusion associated with the sensations. Participant 5 said, “The first time I experienced the coregasm was when I was 7. It was in my gymnastics class and I remember being on the floor and doing the candlestick pose, which is like your arms are on the ground and your legs are up. It’s like a core muscle workout and that’s when I remember first being like ‘oh my god what is this feeling?’”.

Women who recalled first experiencing EIA or EIO at young ages lacked context for understanding their sensations. Some remembered wondering if they “had to pee,” as with Participant 6 who said, “well I was fourteen so I had no idea what had just happened. I thought ‘oh gosh, I must be peeing my pants or something’ so I ran to the bathroom real quick and that wasn't the case and I was like ‘that's weird.’” Participants also described their EIO sensations as feeling novel or unfamiliar—feelings that caught their attention because they hadn’t yet experienced anything quite like it before. Participant 20 said:

Well, my first orgasm that I can remember ever was on my bicycle…I rode my bicycle all over our yard, in our driveway, just around and around all day long. So, my brother and I did this a lot and I felt this sensation that I never felt before or that I never remembered feeling before and I stopped. All of a sudden, I thought, “oh my gosh. I have to pee.” So I got off my bike and as soon as I got off my bike it kind of went away and I was like “oh, I don’t have to pee.” And then I got back on my bike and as soon as I got back on my bike I thought “oh, I have to pee” and then I got off again and I was like “oh, I don’t.” I didn’t know what it was exactly, but I knew that it felt different. And then I later figured out what that was.

Some women whose first EIO occurred in adolescence recognized the sensations as connected to sexuality, especially if they had already experienced solo or partnered sex. Participant 12, for example, described her first EIO experience as occurring around age 15 or 16. By that time, she had engaged in partnered sex and had some context within which to situate the EIO sensations, even though she did not yet recognize the feelings as an orgasmic response:

I knew it was a sexual thing. I knew it was something that had to do with me being sexually aroused, but I was thinking it was something—more of something I had done when I was running. I don’t know. I was just like, “my body is being weird.” [I thought] I had just ran myself too fast …at the time I just didn’t associate…I was thinking, “I am playing a sport; I am not having sex so this doesn’t…so I didn’t think of it that way.”

Women who did not initially recognize their sensations as an orgasm typically indicated feeling that their experiences were physically pleasurable or something they liked. Participant 15 recalled her first EIO experience as positive both in terms of bodily sensations and because of the social context in which it occurred. In her case, her first EIO occurred while she was climbing a rope during her school’s physical education class. She said, “It was actually a really encouraging experience ‘cause I was nearly at the top when I started experiencing these strange things and everybody was [saying] ‘don't stop, keep going, you can make it to the top.’ So, I had to pause for a second and experience what was going on and then I was like ‘okay that was really cool, now I'll keep climbing.’” And even though Participant 17 was not sure what to make of her bodily sensations, they were a regular enough experience for her and she felt good about them:

It was actually probably when I was in seventh grade but I had nothing to compare it to. I didn’t know that it was weird, or I just assumed that it happened to everyone because I played sports so I needed to pass a physical test and I needed to do the bar hang and I used to hang for like fifty eight seconds, almost a whole minute. I would just get a really good feeling but I just assumed…that happened to everyone and I didn’t have anything to compare it to.

#### Understanding Exercise-Induced Orgasm

Despite confusion surrounding early EIA/EIO experiences, participants often described moments of recognition when they first understood the sensations to be genital arousal or orgasm. These “aha!” moments often coincided with becoming interested or curious in sexual activity, as with Participant 19 who connected her first experience of sexual arousal with a partner to her childhood EIO experiences. She said, “it all came together for me, it’s like, ‘well this is the same thing.’ The first time I actually, you know, made out with someone whether it was a boy or a girl back then, I thought, ‘This is the same feeling, how strange.’”

Participant 9 compared EIO sensations with arousal that she had experienced while watching pornography and reading comic books, describing the feeling as “the same ‘itching’ down there.” Others connected their EIO experiences with what they had learned from media, sex education, or peer interactions about arousal and orgasm. Participant 1 described piecing together her EIO experience from childhood games of truth or dare.

I remember Truth or Dare in probably sixth grade and they would dare people to hump. My friend had this dinosaur thing in her front yard and they would always dare this one guy to go hump it and I didn’t understand. I didn’t know, what are you doing to hump it and somehow I learned oh, when you hump it and then you orgasm from it but somehow that got put together and then it just all came together. I was like “oh, okay; orgasming, so that’s what I was doing. That was an orgasm. That’s what that’s been all this time.”

Participant 16—who described her EIO sensations as like a “rushing sensation…maybe pulsating to some extent”—said that “at fourteen I had no idea what that was or how to relate it to anything.” She continued:

I don't even know if I ever really got suspicious that [it was an orgasm] until I had one as an adult for the first time and then kind of correlated and said, “okay yeah, that's what's going on with those.” Up until that point, especially in my youth I was just like, this is kind of a cool sensation. I didn't realize that was a form of kind of masturbating, I guess.

#### Intentional Engagement with Exercise-Induced Orgasm

Many participants described how—after experiencing pleasurable sensations from exercise—they would try to reproduce the experience, sometimes by experimenting with exercise or bodily movement. Most often, this was done in private although some explored with a friend, as with Participant 1 who would seek out these sensations with a friend on the playground:

My best friend and I in elementary, we used to climb up the poles and we would be like, “what is this feeling? What’s going on?” We would both experience it but we didn’t know what an orgasm was. We had no idea. [We’d say], “It kind of tickles but it feels good. I don’t know what it is.” We used to seriously have races to see who could get off first, which is now hilarious looking back on it.

Some recalled these early experiences as forms of masturbation, even though at the time they did not think of their behavior as masturbatory or as sexual. Participant 16 would often seek out EIA/EIO by doing hanging leg lifts in bathroom stalls that had bars installed for accessibility:

They've changed the handicapped bathrooms so they wouldn't be cooperative anymore ‘cause of where they put the bars, but [at the time] they had put one [bar] on each side of the stall, so I could just kind of hang there. Do the hanging lift thing… So, whenever I would be in the bathroom maybe at school or anywhere by myself and I could get into the handicap bathroom, I would just kind of do a few lower abdominal exercises just long enough to generate that experience and then I'd be on my happy way.

Similarly, Participant 18—who first experienced EIO from climbing monkey bars as a child—recalled using a pull-up bar at her parents’ home to reproduce these feelings from the playground. Describing how her parents had installed a pull-up bar over the door, she said “I would do it purposefully…I would do it mainly at home and I think I kind of knew what I was doing.”

### How Exercise-Induced Orgasm Is Experienced

#### How Exercise-Induced Orgasm Feels

Various words were used to describe how EIO felt, including thumping, throbbing, itching, tickling, quivering, and shaking. Perhaps most common, especially among participants who first experienced EIO as a child or adolescent, was the notion that EIO feels like the need to urinate. Participant 1 said, “Just sort of a feeling, like, almost like I needed to pee but, like, in a better way if that makes sense.” Among participants sharing this sentiment, there was consensus that EIO was a pleasurable feeling, as expressed by Participant 20:

It was pleasurable and, when it happened, I thought I had to pee but I knew that it was different than the feeling so it wasn’t—I didn’t feel like I had to pee. I just didn’t know what else it was. So, it definitely felt good. I feel like I kind of, I felt it throughout my body. You know how when you have an orgasm you feel it kind of all over sometimes.

Some described their genitals starting to “throb” or “beat” as with Participant 14, who described it “like a throbbing feeling and a lot of pressure built up, like intense, and then my body just collapsed. Then I felt very tired, out of breath, and I felt very at ease afterwards, as well.” Participant 19, who had experienced EIO in various ways including while horseback riding, described the intensity of the sensations along a continuum. She said:

Like a real rush, an incredible rush. That's probably the only language I can use, a real rush and a couple of times I thought I was just going to pass out which meant that would not be good to fall off the horse. A couple of times as a child I thought I was going to fall out of the swing, you know, swinging really high and that rush and excitement and so I just connected to really high physical sensual pleasure. Maybe it's on some kind of

continuum or something.

A few women also described a buildup to EIO. Participant 14 shared how noticing those sensations will cause her to intensify her exercises:

Yeah, it’s like the rhythm, and then it makes me think about sex. It all happens at the same time. I’m feeling the rhythm, and I’m going and going and going, and then I kind of zone out and I picture myself during this thought, like at the same time. It’s kind of mentally at the same time, but then I start feeling my genitals start to beat, and then that’s when I start to actually go through the arousal process... Beat, like I just feel like thumping kind of... And then I start going harder on my exercises.

Participant 13 also described how the stroke she used would affect the feelings she experienced: “When I’m doing the breaststroke, I feel like the feeling is coming stronger, maybe because the kick…it’s uncomfortable to talk about it… [but] when you open your leg and you do the kick, you have the feelings just coming in. I don’t know why.” These anecdotes reflect how some women progressed from an incidental, uncontrolled EIO experience to a learned, intentional technique.

#### Location of Sensations

Several women identified the sensations as concentrated around the “lower abdominals,” “between the belly button and the pelvis,” the lower abdominal muscles in connection with their leg muscles, or even seeming to “pull” on the muscles around their genitals. Participant 5 said, “it’s definitely like a deep… a deep orgasmic feeling in your—it doesn’t feel like it’s on the clitoris and it’s confusing. When I was younger, [it was] a very confusing feeling because it feels like you’re orgasming in your stomach or in your lower abdominals.” The lower abdominal area was also mentioned by Participant 18 who said, “it starts like lower abdomen, I would say. And then it is kind of a weightless tingly feeling in my legs and then like if I concentrate really hard, it almost feels like the vaginal walls are contracting.” Participant 9 felt the sensations in her lower abdomen but also in her legs, as a pleasant sort of “itching.” She said, “I think my whole legs were really relaxed and my muscles below my belly.”

Participants sometimes associated their EIO with a certain direction of movement within their body, sometimes a “pushing down” sensation. A few women referred to feelings that felt “up and down” in their abdominal area or, for Participant 7, a “radiating downward from below my navel.” Participant 11 perceived her EIO as occurring in a “V-like motion down towards your vaginal area, so from the belly button from left and right sides down towards your vaginal area.” Participant 16 described it as a “pushing down and through… it’s that same sensation of pushing down and through during that whole process…lifting up and bearing down” in the genital area. Participant 12 noted how she could engage with her muscles to either facilitate or prevent EIO. She said, “I don’t know how to explain it, but when I push out that can help me not orgasm, whereas if I pull in and contract them in that helps me orgasm. So, if I am in a [public setting] I try to just push my vagina muscles out.”

Several participants described a progression of their sensations. Participant 7 noted “feeling warm all over” before “I start feeling more down here… I think I feel, like, everything from the waist.” Also noting both the location and order in which her EIO occurs, Participant 11 said “right in between the belly button and the pelvis…the muscles start contracting, and then it kind of just starts tingling, and then the more you do it, it goes down a little bit farther.”

#### Comparison with Sexual Sensations

Many women described EIOs as feeling deep and internal, “like it’s coming from inside the body” (Participant 2), or more similar to orgasms from vaginal penetration as opposed to clitoral stimulation or oral sex. Some compared their EIOs to other sexual sensations. Participant 18 described vibrator use as doing “basically nothing for me…whereas with this [EIO], it’s very like internal, like there is no real external stimulation. I feel it, like, from the inside.” Participant 21 said that her EIOs give her “kind of just like an excited feeling… I am trying to equate it to another experience, because I don't know how to describe that in words, like a feeling you get if you were making out with someone and you think it is going to go further I guess.” Additionally, some women described differences as with Participant 3 who described feeling “unexpected” sensations in the higher portions of her abdominal area:

Participant 3: Not in my sides but it feels—oddly the—I feel it in my genitals and the lower ab portions but also higher up, which is sort of an unexpected thing. It’s almost just like a—burning is the wrong word—it’s like definitely arousal in the genitals but in the higher up section of my abdomen it’s like a quiver—does that make sense like a quivering maybe or like a shaking, even more of a tenderness.

Interviewer: Do you feel that leading up to the orgasm or during the orgasm?

Participant 3: Leading up to. Meaning there’s like this quivering, right. But the actual orgasm, when it gets to actual orgasm it’s in the genitals, lower abdomen, not so much the upper abdomen. But leading up to it yah it’s like this quivering sort of unstable—I mean things you might normally describe an orgasm with but I wouldn’t necessarily experience that same feeling in a sexual situation. It’s an odd—I guess this is kind of odd.

Participant 13 described her EIOs as “more gentle” than sexual orgasms. She said, “I think for [orgasms] during exercise, I feel really relaxed. It’s more smooth. I don’t know how to describe that kind of feeling, but basically, I think if you’re having sex, it’s more sweat. Maybe just because I’m swimming, so my body is really comfy and cold.”

#### Exercise-Induced Orgasm Frequency

EIO frequency varied across participants, from experiencing EIO only a few times to experiencing EIO on a regular basis, including over many years. Some women recalled only a few instances of EIO although they did experience more frequent EIA. Participant 9, for example, described EIA from both yoga and cycling but remembered only two instances of EIO—one during an intensive yoga camp and another on a bike ride after she had “made out” with her biking companion. Participant 12 similarly shared that she experienced EIA during various forms of exercise but only recalled a few instances of EIO.

Others described EIO as occurring “very frequently,” as a regular occurrence during workout routines or however often they performed certain exercises. Participant 7 said, “it wouldn't be every time, but I used to spin like three or four times a week in the mornings, and I would say usually [EIO happened] like at least two [of those] times.” Many recalled experiencing EIO regularly over a period of time, such as Participant 17, who was 23 at the time of the interview and estimated that she had experienced EIO from pull-ups “fifty times more or less” since age 12 or 13.

### Factors Associated with Exercise-Induced Arousal/Exercise-Induced Orgasm

Women identified several factors that they felt caused or contributed to their EIA or EIO, ranging from characteristics of specific exercises to clothing, accessories, and/or menstrual cycle phases.

#### Exercises (Muscles, Pressure, Breathing, and Intensity)

Participants commonly attributed EIO to exercises that required targeted activation of their core abdominal muscles. Exercises frequently related to EIO included climbing, leg lifts, and sit-ups; several others described experiencing arousal or orgasm from cycling, planks/push-ups, and yoga (Table 1).

For women whose EIO experience first occurred during childhood, the exercises were often related to childhood play (monkey bars, climbing poles) or activities required during physical education classes (sit-ups, rope climbing). Participant 1 said:

They were just a series of three levels of poles and we would climb to the top of them.... It was mostly arms and legs. That’s why later when I found out that I was orgasming and that’s what that was, I was like, “how is that even happening when my genitals aren’t really even touching on it.” It was just the act of doing it.

Participant 15 said “[orgasm is] really, really fast with poles. Super easy, like thirty seconds.” A pattern emerged in which women who described greater levels of physical activity tended to describe EIO occurring only after extensive core engagement. Some described EIOs occurring after 200 to 300 sit-ups or crunches or after eight to nine unassisted pull-ups which, while smaller in number, require considerable core strength. Others described engaging in varied forms of physical exercise and, only after sufficient exertion and core engagement, did the EIOs occur. For example, Participant 2 indicated that, as part of playing basketball for her school, her coach had the team working out regularly, doing “crunches, push-ups, that kind of thing.” Following an intense workout, she said “I would go in my room and do 60 crunches and then…yeah, so. [Laughter]…. I think I did straight up 60. I would say I started feeling it (arousal) at 40 or around there and then it would just keep building from that [to orgasm].”

Those who exercised infrequently tended to report EIOs occurring with fewer repetitions (e.g., dozens instead of hundreds) or less time spent in the exercise. Women showed great familiarity with their bodily response in terms of how long it took for a certain exercise to cause orgasm, or how they needed to hold or move their body to experience orgasm. Participant 5 said that her EIOs happen “pretty quickly, pretty suddenly. I could get on the ground and start holding like a plank or start holding my legs in a straight line and get it.” When asked how quickly after starting to ride her bike she experiences EIO, Participant 4 said, “I’d say within twenty minutes, honestly.” Participant 11 described a pattern she noticed related to her EIOs in addition to her sense that the timing of her EIOs might be influenced by her age and/or weight:

I would do three reps of 10, and it would happen within the second to third rep session. Like I would do a second ten, and the other night that’s when I tried it as well, and because I’m older and much heavier, it was actually within almost the third. I couldn’t do it the first couple. I was like, “Okay, maybe this isn’t going to work anymore.” And then I was like, “Let me take a deep breath.” I was trying to make sure all my kids and my dog and everybody was asleep, and I was like, “Let me try one more time.” And then as I started to do the third rep, it started that tingling.

For Participant 6, her routine involved leg lifts that were done “always laying down, never in a captain’s chair” and she would begin to feel aroused after twenty repetitions. “And then all of a sudden, like wait a second,” she said. “It’s like I am definitely into it… and I am getting a little warm and then that’s when I start to notice it.” Participant 17 also described a predictable pattern to her pull-ups leading to orgasm:

I don’t really know how to describe it. It’s just the build-up to orgasm. I know it’s going to happen. Once I feel it building up, I start doing the pull-ups and I just stay around that and I kind of just like wait for it to happen because I don’t know if it would be way too intense if I continued the pull-ups. And so I just kind of let myself hang there but for me…to get the better feeling, to get the best out of it, I lift my legs up to my chest cause when I have my legs up that’s the best feeling, instead of leaving my legs straight down but I don’t know really how to describe the [feeling]…the orgasm and then I just feel my insides throbbing so.

Although EIO felt out of some women’s control, others described being able to relax their muscles, take a break from the exercise, or lie down to delay or prevent unwanted EIO. For others, taking deep, slow breaths enhanced their arousal and facilitated orgasm.

#### Clothing and Accessories

Although some women felt that their EIO experiences had no relation to the kind of clothing they wore (e.g., looser versus tighter fitting), others associated specific fabrics and/or accessories with the likelihood of experiencing EIO. Thinner, more tightly fitting athletic materials including spandex were described by some as increasing friction and thus facilitating EIO, as with Participant 7:

So, you know how there is like exercise stuff that is either wicked thin or it's like designed to be that it feels very thin and light and those type of materials, it's definitely easier for me. Whereas like if I am going to wear cotton, because I do have some like cotton exercise pants, it tends to be a little harder in that. I feel like it's thicker and heavier and, yeah, I’ve definitely noticed it being more difficult. It's very easy for me to start to be along that path when I am wearing the thinner material.

This was the case for women who described clothing as influencing their EIO, including Participant 13, whose EIO experiences occurred while wearing tight-fitting one-piece swimsuits while swimming. Another participant noted that clothing just needed to feel comfortable (as opposed to more restrictive clothing like denim) for EIO to occur. One woman said that EIO happened more easily when she exercised without clothes at home. Participant 7 described that EIO became more consistent after having her clitoral head pierced: “I am confident that it has intensified from that piercing. The arousal I had was inconsistent, so it wasn't like every time. I would have it occasionally, but I would notice it and like, ‘Whoa. Okay. That's nice.’ But definitely with the piercing, it makes everything far more sensitive for me down there because it sits on it.”

#### Menstruation and Menopause

Several women had observed greater ease or difficulty of EIO in relation to their menstrual cycle. Participant 14 said, “It probably would be after my menstrual cycle, because that’s when I’m probably the most aroused, or right before my menstrual cycle. But if it’s not two or three days before or two or three days after, I probably wouldn’t be able to tell. I know that’s my most aroused times.” For Participant 12, her EIO experiences were sometimes made easier by wearing a tampon while exercising: “The muscles inside your vagina, I think they contract around whatever is in there. [Laughter]. So, when I do certain exercise, every now and then I won’t even notice that I am doing it but I will be doing Kegels…. when you have a tampon in and you are doing those, I don’t know; I think it obviously arouses your body.”

Participant 19 said that she had noticed EIO in relation to her menstrual/ovulatory cycle, when she was younger, but she also spoke about EIO changes she noticed after menopause. This participant still perceived EIO as aligning with what she described as her “ovulation cycle,” even after menopause, and that her understanding of her less frequent EIO experiences after menopause were less associated with biological/hormonal changes, and more associated with changes to her physical activity and interests:

I would say the ovulation cycle would really stimulate it and I think that doesn't even go away after you stop everything in menopause....Well, I don't do the same things. I can't ride horses anymore because of my back. I don't do the same things, but I think, when my granddaughter was little, I used to swing with her, I'd still get that rush…[and] a lot of the same feelings. It's lessened, it's lessened and I think with age your interests change…[The] focus is a little different but I can still get rushes sometimes doing certain things, like I have a riding lawnmower, which I love to mow the lawn and that can happen on a lawnmower.

### Communication About Exercise-Induced Orgasm

Women described experiences with and motivations for talking (or not talking) about their EIO experiences with people in their lives. Participant 14 expressed feeling alone in her experience of EIO, and anticipated judgment related to sexuality if, as a child, she had told her mother about what she had felt:

I knew it was probably something to do with sexual thoughts or anything like that, and at the time, of course that was nothing you wanted to tell your mom. So, I didn’t know if I told her that and she would be like, “So you’re sexually active or you’re having thoughts about sex?” I just knew it wasn’t normal. I just didn’t feel like people experienced that, so it was just like my own secret.

Participant 1 also did not discuss EIO with her parents. “Part of me thought I shouldn’t, because then there was on our staircase—at the top of it there was a little railing part and I would hump, climb on that thing too. [Laughter]. But I would never do it if my parents were around and I never told them what I was doing.” Others shared that, because they understood EIO as associated with their vagina or sexuality, they shouldn’t discuss EIO with others. Participant 3 said that, because she hadn’t yet had partnered sex when she first experienced EIO and therefore didn’t connect her or her friend’s exercise experiences with sexual feelings, she would have just described it “as it feels really good, our body just feels good.” However, noting that some of the girls on her team had had sex before, she said that one older girl said, “‘You guys are totally having orgasms during those hanging knee ups. I know what’s happening.’ And I was a little bit embarrassed about it but not—that doesn’t really freak me out, talking about that. But we would say, ‘It feels really nice.’ Or this might sound weird but sort of an exciting feeling.”

Some sought validation through connecting with others about shared EIO experiences. In recalling how she and her elementary school friend would discuss their experiences, Participant 1 said, “we were trying to see if we were both feeling the same thing and saying that it kind of feels like you have to pee but it kind of tickles but it feels good…We were the only people that ever talked about it.” She said she and her friend noticed “another girl that used to do it too at recess, but we weren’t really friends with her” and so they didn’t discuss their sensations with her. Participant 1 shared that she and her friend, as children, had a sense that they should cloak their activity, noting “We would actually act like we were climbing to the top of the pole to try to disguise what we were doing but [the other girl] would just sit at the smaller pole and just pull herself up repeatedly on it.*”* Here, she contrasts her and her friend’s awareness that they should hide their public pursuit of EIO with the other girl’s potentially more conspicuous actions.

Participant 15 described asking “at least 20” friends about the experience to see “if this happens to anybody else,” but failing to find others who had similarly experienced EIO. However, in sharing her experience with EIO she said her friends generally reacted by saying, “‘that's so cool’ or ‘that's so weird…I've never heard of that before’ is probably the most common thing they say.”

For some, a gynecologist had been a source of validation or education about EIO, and a figure with whom some participants felt comfortable discussing their EIO experiences. Participant 11 said, “I didn’t know why, but I had never discussed with anybody but my OB, but I had done them just to be doing them lots of times, because [I was] kind of embarrassed to talk about it with anybody, but I’m older now and not embarrassed.” Participant 6 shared that it was “really easy to talk to [my gynecologist], and I asked her and she said, ‘Yeah, that’s quite normal. Unless you’re spasming out of control in the middle of the gym it should not be a problem.’”.

### Feelings About Exercise-Induced Orgasm

Participants’ experiences with, and feelings about EIO, varied. For some, EIO was embarrassing, distracting, or associated with shame, whereas others associated more positive feelings with their EIO, including enjoyment and empowerment.

#### Embarrassment

A common emotion that participants expressed, at least when they first began experiencing EIO, was embarrassment. Some worried about others finding out or noticing their EIO experiences when they occurred in public, such as whether they made audible noises during the orgasmic buildup or whether they had noticeable vaginal discharge. In other words, participant experiences reflecting embarrassment were characterized by concerns about the social environments that they experienced EIO/EIA within. In response, some women developed strategies to keep their EIOs private, such as trying to stay quiet or keeping their face expressionless. One woman who experienced EIO from climbing poles on playgrounds said that she made sure to only climb “for a very short period of time, not to be a sketchy adult there.”

Participant 13 described how she would deal with high levels of sexual arousal while exercising at the gym:

Participant 12: Well, I have never ever told anyone this and I will never tell anyone this besides you guys, but for this sake, there have been times and I would feel really embarrassed, but there have been times where I’ve either had to leave the gym or go to the bathroom and just relieve myself. [Laughter]

Interviewer: Masturbate?

Participant 12: Yeah, just so I can get back to whatever I was doing, because sometimes it can get really to where I am like I need sexual relief or else I am going to have to quit. So yeah, that is what I would do or I think I would just get so embarrassed thinking that people know what I am thinking or feeling that that alone would just get me off of it and I would just get back to exercising.

Participants described strategies for avoiding EIO by opting for exercises that would not produce arousal, or by masking their EIOs by controlling their facial expressions:

I was doing some personal training and I realized at some point that it could actually be very awkward if someone was having me do pull-ups as part of a training regimen and I couldn't stop it because I couldn't imagine it not happening even if I didn't want it to. I think that I could stop it or like not have my legs be in the right position, but I'm not sure. So I ended up not—I said two things to the personal trainer which was that my arms were sore from biking. So, I didn't actually get a chance to test that out. We had been talking about what to focus on and I was saying like, you know one piece of gym equipment is a pull-up bar and he was like, "Oh, that's great, we can do some of those." I was like, actually, uh-oh. (Participant 15)

At that point it's definitely hard because I have been working out and I'm aroused now and I am getting stimulated and I am trying to relax my body simultaneously while working it. And it's this big mental thing going on and it's like, “You need to relax. Stop feeling this way.” It's like the whole mental chatter... I feel like I am a very expressive person, so I tend to try to look very, I feel like my facial expression is very serious to where I am just like, like I am trying to concentrate and like either look like maybe I am forcing my facial expression to look like I'm trying really hard with my abs so it doesn't look like I am being aroused. I mean, because I feel like I am probably expressive. (Participant 7)

#### Shame

For several participants, negative feelings about EIO were rooted in shame related to sex and sexuality as well as discomfort with experiencing and talking about these topics. Different from embarrassment (described earlier), feelings of shame tended to take on a sense that they had done something wrong or were, in some sense, bad. Participant 13, who had been raised in China and felt family-influenced shame around masturbation and sexuality, shared her concerns that others might notice that she was feeling aroused and/or orgasmic while swimming:

Participant 13: When I feel embarrassment, I would just like, “Maybe I’ve done something wrong. Maybe I should stop it.” It’s just really awkward feeling and make me kind of feel kind of shamed. I don’t know, it’s just a feeling. My face kind of start flush, and then, I don’t know, I’m so afraid people are going to see my face flushing.

Interviewer: While you’re swimming?

Participant 13: Yeah, because my skin is kind of pale, so it’s really obvious. People will come and ask, “What’s going on?” I don’t really want to answer some kind of question like that during that time.

Interviewer: Although before you were saying you weren’t really worried about other people noticing.

Participant 13: I know, but when you get up to the water, I would feel shame afterwards, so I was thinking about that all day because it was just really awkward. I would get really shamed. People would say, “Why are you so quiet? Why is your face flushing?”

Participant 3 said, “Because of the body part, the vagina is not something I would really talk about with my (male) coach. In the same way that I wouldn’t talk about periods with my coach or sore boobs or anything like that. It was all connected to the same; I can’t talk about this with a boy.”

Sometimes shame was culturally based. Participant 21 traced her feelings of shame related to her EIO experiences back to her Baptist education.

Interviewer: So this has happened on (bike) rides that you have gone on with other people?

Participant 21: Yes, unfortunately.

Interviewer: Why unfortunately?

Participant 21: I don't know, because it is not something I want to be doing when I am working out I guess... I don't know, to me like maybe it is just the same feelings of like it doesn't feel appropriate I guess.... I don't know… I think a lot of it is to do with, like, I am not like free like, I feel like really constricted about talking about those things, I think because of the way I was raised, it is not supposed to be talked about openly. I went to school from kindergarten to 7th grade at Baptist school and it was very, very, I don't know, like it was very, what is the word? Do you get my gist? It was even further than - radical, I would say radical. Like “if you do this you are going to hell.” I think I get that still kind of, even though I know it is okay, I think that kind of stays there.

#### Distraction

Two participants indicated that EIO was something they would rather not focus on during exercise, but feel they have little control over, perhaps even akin to intrusive thoughts. Participant 21 said:

I mean I have always enjoyed, pleasurable, exciting, all the good ones, you know for sexually induced orgasms. For cycling, I don't know. I just can't shake the feeling that that is like, “Why are you being so weird?” You know what I mean? I don't know. I mean it is still pleasurable but it is not like…. Maybe I, like, dull the intensity myself on purpose too, because I am like trying not to focus on that, I am trying to focus on my workout.

For Participant 3, EIO felt inextricable from sexual arousal, leading to thoughts about people and relationships which felt “aggravating” for her and competed with her desire to focus only on her exercising:

If you think how—if I really just wanted to have a good workout and not feel that orgasm or not feel any arousal at all, just completely separate it from that, I don’t feel like I would have that sort of control, which I don’t really like that the more I think about it. I think because there are times where I just want to focus on working out for the sake of the workout and for no other reason. To better myself physically. And I think when these sexual arousal and orgasm feelings start coming into play it’s a diversion mentally... if I’m training for something I don’t want to be thinking about anything else other than this run or this workout. And to have feelings that are connected to sex brings other thoughts and feelings and thoughts of other people or other times that you’ve felt like that. And not that that’s necessarily a bad thing but sometimes I just don’t want—it’s hard, it is difficult to experience the feeling of an orgasm and not think of sexual partners. So sometimes I don’t want to be focusing on relationships or boys.

#### Enjoyment and Acceptance

Many women expressed mixed feelings about EIO and/or an evolution in how they’ve felt about EIO since their first experience. Often, women who initially felt confused, ashamed, uncomfortable, or embarrassed ultimately came to accept, or even embrace, EIO. Although a handful of participants felt that EIOs could be a nuisance or should be avoided in certain situations, others scheduled workouts to experience EIO. Participant 19 said, “I liked it. It felt great once I got used to it. You know, it was kind of weird at first and then I thought, ‘I wonder if everybody does this, if they fall off swings.’” Participant 20 said, “I like it now. When I was twenty, I don’t think I liked it, but I’m much more comfortable with my body…and I think I’m not…you know, now that I know that it’s something that happens to people, I’m not afraid of it or worried by it, I guess. So I like it, I think it’s neat.” Participant 5 said, “A lot of times I would receive it during workouts so I felt like I had to make it look like I wasn’t coregasming at the gym. And now it’s like I know it can happen and I can also stop it but sometimes I just let it happen at the gym, I can control my face and make it look like it’s not happening and it’s the best way to end your workout.”

Participant 13, who initially experienced shame about EIO, described her path to embracing orgasms as part of exercise: “First I was really shameful. I never thought I would have feeling like that… I was a really young age. I’m not really clear, what should I do? It’s just really awkward feeling just coming from my body, so I feel shameful. But then it was like, ‘Oh, whatever.’ I don’t care anymore. I just go for it because it feels so good.”

For some, EIO was an explicitly positive experience, and one which was seen as an added benefit to or motivator for exercise. Participant 15 said, “I mean I could probably climb without having orgasms, but I mean really why would I want to?” Similarly, because “it’s hard to pull [100 reps] off,” Participant 16 described orgasms as a motivator. She said, “I'd get to the point where I'm like “well I've done this many, I'm just going to keep going just for the added perk at the end of the 100 reps” or something… but I was getting great abs at the same time, so I couldn't really argue with that.” Others shared that EIO made them feel as though they could take control of their sexual pleasure. Participant 11 said: “My girlfriend’s like, ‘I can’t believe you would just talk about that.’ And I said, ‘You know what? It’s natural.’ We as women need orgasms to release stress and tension in our everyday lives. And if you’re not getting the attention from your husband that you think you should have, there are other ways to make sure that happens. That’s the way I feel.”

Participant 14 also described EIO as a way to release sexual tension as well as a motivation to exercise. She said:

If I am very aroused on my own and just like, “This is not happening” or “I don’t want to do this” then I’ll probably go exercise. And then, “Well, I can do it on my own.” Yeah, I think it does motivate me at times when I feel my body’s intense anyways, I’d probably go exercise just because I know that it’s a way that I can release it…once I noticed that I could [have an EIO], I think I started exercising more, just because I knew how I could probably make myself orgasm. I thought it was very cool when I first did it… Because I did it on my own…it was kind of like another secret, like, ‘I wonder if other people do this on their own?’ And it was just cool because I had felt a whole different experience, and it was just me experiencing it. I didn’t need anyone to help me. I just felt really at ease, so it was really cool.

### Intersection Between Exercise-Induced Orgasm, Sex, and Masturbation

Some women described EIO as gradually becoming part of (or even essential to) their sexual repertoire. Several participants recalled using various exercises to enhance pleasure from masturbation throughout middle and high school. Participant 11 shared that she sometimes preferred EIO to manual masturbation.

At first, I was just focused on the exercise and getting the exercise done. But when I figured out what was happening and doing it for the end result, because masturbating sometimes is too quick for me. Sometimes it’s too quick, or multiples is good, but for the exercise I feel like I’m getting two benefits. I’m exercising, getting some sort of muscle worked, plus I’m getting happiness at the end. It’s the best of both worlds.

Participant 5 expressed that her experiences with EIO both taught her about her sexual functioning and improved her partnered sexual encounters:

I don’t think I purposely meant to do it I just think that I know being able to coregasm at such a young age I was able to really learn about my body the way it works, what positions I can get it in, how fast it will take me. And so when I started having more consistent sex I was able to work my body and kind of push myself and tense my muscles to receive a stronger or faster orgasm. And it’s been great because like I’ve been able to, like my partner and I were able to most of the time receive orgasm at the same time because I can purposely be like “oh you’re going, okay” and really tense my muscles hard and receive an orgasm. He’s always like “well is it me or is it your coregasm?” and you know at the end of the day it doesn’t really matter. It’s probably a little bit of both; it’s probably tensing my muscles and you at the same time.

Others had tried to replicate similar muscular contractions in partnered sex, however, the resulting orgasm was less intense than her EIOs. However, Participant 15 noted that some of her best sexual experiences were those that she incorporated pull-ups into, stating “foreplay is somebody telling me ‘okay, why don't you go, like, do some pull-ups now?’” The notion that the ability to experience orgasm through exercise could increase pleasure in partnered sex was common across participants, with some even stating that EIO had been the only way they were able to orgasm. Participant 15 said, “[Having orgasms from climbing and pull-ups] has become much more important with partners recently because I've never actually been able to have an orgasm without doing that until I recently got a toy that works really well so that's cool.”

## Discussion

The present study extends the existing and emerging literature on exercise-induced arousal and orgasm. We provide detailed qualitative interview data on women’s experiences with EIA/EIO, sharing rich insights into women’s lived experiences of how they recall first experiencing EIA/EIO, how they interpreted these feelings of genital arousal and orgasm during play and/or exercise, and how these experiences have felt to them at different stages of their lives. These are novel data that help to grow the field’s understanding of EIA/EIO. Further, our data provide four key observations to extend understanding of EIO and of orgasms more generally: EIO as a component of embodied sexual learning during childhood and adolescence; EIO as a motivating and pleasurable outcome of intentional behaviors; EIO as a focal point for the boundaries of public and private spaces that regulate and sometimes punish sexualized bodies; and the complex psychophysiological phenomenology of orgasm that includes non-sexual and non-genital contexts. Each of these observations is discussed in detail below.

Few studies address orgasm capacity in childhood and early adolescence although about 7% of 18-year-old Swedish women reported masturbation to orgasm between ages 6–10 years (Larsson & Svedin, 2002). Consistent with this observation, and with prior research (Herbenick & Fortenberry, 2011; Herbenick et al., 2021), first EIO often occurred in childhood or early adolescence. Thus, our data provides additional evidence for functional capacity for both genital and non-genital orgasm during childhood, as well as experiences for embodied learning that may influence interpretations of subsequent sexual experiences (Tolman et al., 2014). Embodiment may help explain why several participants linked EIO sensations to those of urination. Note that “orgasm” here is a generic term applied to experiences reported by and recalled by adults, whose experiences with their bodies help them interpret and understand these childhood experiences as orgasm. The degree to which these recalled childhood experiences replicate the neurophysiologic parameters of genital arousal and orgasm among adults—for example, increased genital blood flow and post-orgasm increases in prolactin—is unknown. Indeed, it is not even clear whether and to what extent adults’ experiences of EIO track with the neurophysiological markers of sexual arousal and orgasm; this should be investigated in future research with adults. Future research might also examine whether and to what extent people who experience EIA/EIO differ from those who do not in various personality traits or psychosexual variables, as has been examined in prior research on people’s experiences with orgasms during sex (Harris et al., 2008).

Second, although participants who experienced EIO as children didn’t initially interpret their sensations as sexual (and indeed they weren’t) or as orgasmic (given their lack of knowledge, at early ages, about what an orgasm was), many described a desire to reproduce the pleasurable feelings. These interests in re-creating the bodily sensations led many participants to engage in various exercises or forms of play that “worked” to produce these sensations. This observation adds insight into the ontogeny of sexual pleasure during adolescence, when development of specific sensory capacities aligns with hormonal influences on cognitions and social influences on expectations for sexual pleasure and specifically sexual interpretations of embodied sensations (Saliares et al., 2017). Also, although some women had to overcome feelings of confusion, embarrassment, or even shame related to their EIOs, most were able to enjoy their body’s ability to experience arousal or orgasm from exercise. In some ways, this is similar to how many girls and women describe having to overcome negative, or mixed, messages about masturbation to eventually embrace sexual pleasure (Bohmer et al., 2022; Thorpe et al., 2023). Further, most were able to explore their arousal and orgasmic response on their own terms and at their own pace—quite different than many adolescent and young adult women’s experiences with partnered sex, which is too often marked by sexual pressure, coercion, or a focus on their partner’s sexual pleasure (Armstrong et al., 2012; Blythe et al., 2006; Chadwick & van Anders, 2022; Chadwick et al., 2019).

A third notable finding is that many participants’ early EIO experiences—for some, their first orgasms of any sort—took place in public spaces. As a result, their EIO experiences were sometimes associated with shame, embarrassment, or anxiety about observation by others. These experiences illustrate the complex ways physical activity invokes the strict public/private boundaries related to sexual bodies and sexual comportment (Fortenberry & Hensel, 2022). Vigorous activity in the form of play, social activity in the form of athletics, and exercise as a form of leisure are regularly enacted in shared public spaces like playgrounds, parks, fitness and yoga centers, and gyms, as well as sidewalks and streets. Orgasm, in its associations with sexual activity, is a quintessentially private experience regulated by social proscription related to sexual modesty as well as legal prohibition of public sexual activities (Fortenberry & Hensel, 2022). The social aspects of these orgasm experiences could be positive (especially when the person didn’t yet connect it to sex or when they felt no one could tell what was happening). And indeed, some described childhood EIO experiences that occurred around classmates at their school physical education class or on a playground and these were often associated with friendship, encouragement, exploration, and playfulness. However, others described feeling self-conscious or ashamed, even if their EIO was undiscerned by others.

Finally, our findings provide a range of detailed descriptions of the subjective experience of EIO. These generally align with descriptions of orgasms associated with orgasms from genital stimulation (King & Belsky, 2012; Mah & Binik, 2002) although women with EIO experiences also sometimes noted interior abdominal sensation, with less emphasis on genital/clitoral sensation. Although EIO is among the broad set of orgasms not explicitly associated with sexual contexts and genital stimulation, the subjective experience for these orgasms has not been described (Herbenick et al., 2018). Future research might explore EIO in the context of the Orgasm Rating Scale (Mah & Binik, 2020) to better understand, and potentially differentiate between, orgasms in sexual contexts compared to orgasms from exercise. Further, women described experiencing EIO from diverse forms of exercise, and especially those that are demanding of the core abdominal muscles—again, consistent with prior research (Herbenick & Fortenberry, 2011; Herbenick et al., 2021). Many participants seemed able to pinpoint a certain point during exercise when EIO was likely to occur, usually after a certain number of repetitions of an exercise, or after achieving a specific intensity, which supported their understanding of their bodily responses.

### Strengths and Limitations

A strength of our research is that the interviewers (a sexuality researcher and personal trainer) brought complementary expertise to the study. Our study also had several limitations. First, although we present women’s descriptions of how EIOs feel to them, a mechanistic explanation for how EIO occurs remains unknown. We don’t yet know how it is that exercise leads to orgasm for nearly 10% of the US adolescent/adult population, but this seems important to know both because some people wish to experience this and some people wish to avoid EIO. Indeed, some women described feeling bothered that they could not prevent EIO from occurring, which suggests some similarity with the more common male experience of ejaculatory inevitability. Subsequent research is needed to understand how arousal and/or orgasm may be caused during physical exercise and should also assess people’s EIO experiences in relation to lubrication, engorgement, and/or erection.

A second limitation is that we only recruited adult women, most of whom were young (between the ages of 18–26). Subsequent research should engage men and gender diverse participants, as well as those of older ages to examine the extent to which EIA/EIO may change in relation to pregnancy, the postpartum period, menopause, or other life changes. We also did not systematically ask participants about their racial, ethnic, or cultural identities or their sexual orientation identity, although these were often volunteered as part of their narratives. It is possible that people’s experiences with EIO/EIA may vary based on these and other demographic characteristics, thus warranting subsequent research in more focused populations. Further, although a few participants described some health challenges, they were a mostly healthy group of women who could exercise without supervision or assistance. Subsequent research should examine EIO in the context of disability, including the experiences of those who may need to exercise in the presence of physical therapists, recreation therapists, or personal care attendants.

### Implications

Our findings have implications for sexuality educators, who might expand the ways in which they talk and teach about orgasm—situating it as a neurophysiological experience and not necessarily a sexual one. Our study also has implications for clinicians, who might ask their clients about their experiences with arousal or orgasm from any sources (not just from sex); acknowledging exercise as a potential source of such sensations may stimulate conversation and openness about EIO. Clinicians might also note the extent to which many of the women in our study felt unable to speak about their EIOs, often lacking an understanding of their bodily responses, feeling “weird” for having EIO, or shame due to family or cultural sexual taboos. Finally, our study has implications for coaches and personal trainers who need to be aware that some people may be experiencing EIA/EIO during exercise—often unintentionally and without an ability to control their bodily response. It may be helpful to give people permission to abstain from certain exercises, or to modify their approach to such exercises, to make exercise more accessible to those who experience EIO. Since leading research on EIA/EIO, the first author has heard from elite athletes, as well as the parents of high school and college athletes, who have found that EIO interferes with their (or their adolescent/young adult child’s) exercise training. The first author has also heard from military physicians who have struggled with finding ways to help some of their patients avoid EIO so that they can successfully pass military fitness tests. Subsequent research on how EIO works, as well as how it might be managed, will have important implications for people’s fitness and, for some, their livelihood.

### Conclusion

In conclusion, the present research adds to a growing body of research on exercise-induced arousal and orgasm. We found that EIA/EIO was commonly experienced in childhood and adolescence and often took time to understand as sexual or orgasmic. Over time, women generally organized their exercise experiences into their broader sexual repertoire and often came to embrace their experiences.

## Data Availability

Not available, given considerations related to raw qualitative data that could be identifiable.
